# Supplementary material for: Resolving the full spectrum of human genome variation using Linked-Reads
Source: Genome Res. 2019 Apr;29(4):635–45. doi: 10.1101/gr.234443.118 (PMC6442396; doi:10.1101/gr.234443.118)
Supplement: Supplemental Material [file supp_gr.234443.118_Supplemental_longranger-2.2.zip › longranger-2.2.tar.bz2!/lr2.2-public/lib/stan/cmdstan/stan/doc/api/html/common_8hpp.html]

Stan: src/stan/common.hpp File Reference


|  |  |
| --- | --- |
|  | Stan  2.5.0  probability, sampling & optimization |


- Main Page
- Related Pages
- Namespaces
- Classes
- Files

- File List
- File Members

All Classes Namespaces Files Functions Variables Typedefs Enumerations Enumerator Friends Macros Pages

- src
- stan

common.hpp File Reference

`#include <stan/common/command.hpp>`  
`#include <stan/common/do_print.hpp>`  
`#include <stan/common/init_adapt.hpp>`  
`#include <stan/common/init_nuts.hpp>`  
`#include <stan/common/init_static_hmc.hpp>`  
`#include <stan/common/init_windowed_adapt.hpp>`  
`#include <stan/common/print_progress.hpp>`  
`#include <stan/common/run_markov_chain.hpp>`  
`#include <stan/common/recorder.hpp>`  
`#include <stan/common/sample.hpp>`  
`#include <stan/common/warmup.hpp>`  
`#include <stan/common/write_error_msg.hpp>`  
`#include <stan/common/write_iteration.hpp>`  
`#include <stan/common/write_iteration_csv.hpp>`  
`#include <stan/common/write_model.hpp>`  
`#include <stan/common/write_stan.hpp>`

Go to the source code of this file.


---

[ Stan Home Page ]

*© 2011–2014,
Stan Development Team.*
